# Molecular docking analysis of phytochemicals with estrogen receptor alpha

**DOI:** 10.6026/97320630018697

**Published:** 2022-08-31

**Authors:** Misbahuddin M Rafeeq

**Affiliations:** 1 Department of Pharmacology, Faculty of Medicine, Rabigh, King Abdulaziz University, Jeddah - 21589, KSA

**Keywords:** Breast cancer, estrogen receptor, tamoxifen, phytochemicals

## Abstract

Breast cancer (BC) is linked to estrogen receptor alpha (ER-α) positive. Tamoxifen and other estrogen selective modulators have proven to be beneficial in slowing the progression of ER-α BC. However, tamoxifen resistance emerges as a result of long-term treatment and cancer development. Therefore, it is of interest to document data on the molecular docking analysis of phytochemicals targeting with Estrogen Receptor-alpha. The screening of the phytochemicals from the ZINC database (a total of 87133 compounds) against ER-α protein was completed. We show that ZINC69481841 and ZINC95486083bind strongly to ER- with binding energies of 10.47 and 11.88 Kcal/mol, respectively, which were significantly greater than the control compound (−8.32Kcal/mol). ZINC69481841 and ZINC95486083 were found to bind with the key residues (Leu387, Arg394, Glu353, and Thr347) of ER-α protein. Data shows that the lead compounds (ZINC69481841 and ZINC95486083) have an acceptable range of ADMET and drug-likeness properties for further consideration in drug discovery.

## Background:

Cancer is defined as a persistent aberrant cell condition or a fatal disease characterized by immortality and uncontrolled cell proliferation. Cancer cells can be invasive, aggressive, and metastatic, spreading to several organs. Breast cancer (BC) is very heterogeneous in character and disrupts the function of normal mammary epithelial cells. BC is the most prevalent noncutaneous malignancy and the main cause of cancer-related mortality in women globally [1]. It affects more than one in every 10 women globally [2]. One of the primary causes of BC is excessive estrogen production. The estrogen receptor (ER) is a nuclear receptor that is efficiently activated by binding to 17β-estradiol legend and is also known as estrogen. ER-α and ER-β are naturally present in humans and have a role in the regulation of many physiological processes including cell growth and differentiation; among them, ER-α is mostly expressed in the mammary gland and uterus [3]. In women, ER exhibits an important role in BC apoptosis, inflammation, proliferation and differentiation. ER-α is widely known for its role in immune surveillance, apoptosis resistance, metastasis, and cell proliferation [4,5]. The overactivity of estrogen hormone may result in the multiplication of ER-α, which may contribute to the maintenance and growth of BC types. Nowadays, phytochemicals are being studied for their potential use in modern medicine and contribute vital role in the synthesis of a wide range of therapeutic agents [6]. Phytochemicals have been shown to have a variety of beneficial effects on human cancer models [7-9]. Computer-assisted drug design methods have greatly aided in the efficient processing of cheminformatics and bioinformatics information, therefore speeding early drug development efforts through rigorous molecular docking simulations [10-15]. Therefore, it is of interest to document data on the molecular docking analysis of phytochemicals targeting with Estrogen Receptor-alpha.

## Methodology:

### Protein preparation:

The 3D crystal structure of ER-α (PDB ID: 3ERT) was accessed from the protein data bank (https://www.rcsb.org/structure/3ert). As 3ERT is a homo2-mer structure, one chain was removed and a monomer was used for the docking analysis. The protein preparation was done with the help of Discovery Studio's protein preparation tools.

### Library preparation and virtual screening:

Phytochemicals from a commercially available ZINC database (natural product + in vitro) (https://zinc.docking.org/substances/subsets/natural- products+in-vitro/) were used (a total of 87133 compounds) for virtual screening in this study using the PyRx 0.8 program. PyRx was employed to prepare the whole ligands before molecular docking to get various binding conformations with the least binding energy (BE).

### Molecular docking:

Docking tools like AutoDock and others have made it feasible to quickly screen ligand molecules using posture prediction and ranked list outputs [16,17]. Molecular docking of lead compounds were performed using AutoDock 4.2.Grid points were set as 40 x 40 x 40Å with the spacing of 0.375 Å, and X, Y, and Z values were kept as 27.432, -2.033, and 26.269, respectively. Other parameters in the docking procedure were set as default. For each docking system, 100 independent docking runs were performed. The best postures of each lead compound were chosen based on the lowest BE once the docking calculations were completed.

### Pharmacokinetics and toxicity estimation:

Swiss ADME (http://www.swissadme.ch/) [18] and pkCSM (http://biosig.unimelb.edu.au/pkcsm/) [19] web tools were utilized to predict the physicochemical characteristics, pharmacokinetics, drug-likeness and toxicity properties of the ZINC69481841 and ZINC95486083.

## Results and discussion:

BC is one of the most common types of cancer in women. Notably, ER-α positivity accounts for 70% of all BC diagnoses, makes it a key therapeutic target. Prospective therapeutic compounds that modulate ER-α are now being explored for the prevention and treatment of a wide range of pathological disorders including the BC [3]. This study screened a library of phytochemicals from the ZINC database against ER-α protein. Among them, lead compounds ZINC69481841 and ZINC95486083 were found to strongly bind with ER-α. Figure 1(see PDF) depicts the two-dimensional structures of lead compounds.

ZINC69481841 was observed to interact with Met343, Thr347, Leu349, Ala350, Glu353, Trp383, Leu384, Leu387, Met388, Leu391, Arg394, Phe404, Met421, Ile424, Leu428, and Leu525 residues of ER-α (Figure 2 - see PDF); while Met343, Thr347, Leu346, Leu349, Ala350, Glu353, Trp383, Leu384, Leu387, Met388, Leu391, Arg394, Phe404, Glu419, Met421, Ile424, Leu428, Gly521, and Leu525residues were found to bind with ZINC95486083 (Figure 3 - see PDF). Leu387, Arg394, Glu353 and Thr347 have been determined as active site residues of ER-α protein [20]. Interestingly, ZINC69481841 and ZINC95486083 were also found to bind with these ER-α protein residue.

The BE values for ZINC69481841 and ZINC95486083 with the ER-α were observed to be -10.47, and -11.88 kcal/mol, respectively, while the inhibition constant were 4.57 and 3.21 µM, respectively (Table 1 - see PDF). Tamoxifen is an antiestrogen [21] that was used as a control compound in this study. BE of tamoxifen with ER-α was found to be -8.32 kcal/mol. The H-bond contributes to the stability of the "inhibitor-protein" complex and aid in determining the inhibitor potency to the target protein [22]. Glu353was the common H-bond interacting residues of ER-α with ZINC69481841 and ZINC95486083 (Figure 2 & Figure 3 - see PDF). Further, in order to get a better picture of ER-binding residues with the lead compounds, we analyzed ER-binding residues with its co-crystallized ligand (PDB ID: 3ERT) [23], which showed that Met343, Leu346, Thr347, Leu349, Ala350, Asp351, Glu353, Leu354, Trp383, Leu384, Leu387, Met388, Leu391, Arg394, Phe404, Met421, Ile424, Leu428, Gly521, His524, and Leu525 are important in interaction with its co-crystallized ligand (Figure 4 - see PDF). Consistent with this, Met343, Thr347, Leu349, Ala350, Glu353, Trp383, Leu384, Leu387, Met388, Leu391, Arg394, Phe404, Met421, Ile424, Leu428, and Leu525were the common interacting ER-α residues with the ZINC69481841 and ZINC95486083 as well as the co-crystallized ligand (Figure 2, Figure 3 & Figure 4 - see PDF).

Molecular docking has shown to be a useful method and has been utilized in numerous inhibitor discovery investigations to identify potential inhibition mechanisms and to illustrate the nature of molecular interactions between an active molecule and its target [24-26]. In docking studies, the strength of interaction between ligand-protein complex is assessed in terms of BE, and the lowest BE (more negative) is the result of the ligands efficient binding to the active site of the target protein [27]. Accordingly, lead compounds ZINC69481841 and ZINC95486083 showed strong binding (lower BE) with the ER-α than the reference compound (tamoxifen), suggesting that these compounds could be utilized as an inhibitor of ER-α to fight the BC. In silico pharmacokinetic and toxicity prediction analysis determines that lead compounds (ZINC69481841 and ZINC95486083) have an acceptable range of ADMET and drug-likeness properties (Table 2 & 3 - see PDF).

## Conclusions:

We describe the molecular interaction of phytochemicals with the estrogen protein.ZINC69481841 and ZINC95486083 show strong binding with the ER protein as well as satisfied adequate ADME criteria for further consideration in drug discovery.
